# Optimal growth temperature of Arctic soil bacterial communities increases under experimental warming

**DOI:** 10.1111/gcb.16342

**Published:** 2022-07-24

**Authors:** Ruud Rijkers, Johannes Rousk, Rien Aerts, Bjarni D. Sigurdsson, James T. Weedon

**Affiliations:** ^1^ Amsterdam Institute for Life and Environment, Section of Systems Ecology Vrije Universiteit Amsterdam Amsterdam The Netherlands; ^2^ Microbial Ecology, Department of Biology Lund University Lund Sweden; ^3^ Faculty of Environmental and Forest Sciences Agricultural University of Iceland Borgarnes Iceland

**Keywords:** Arctic, climate change adaptation, microbial communities, soil warming

## Abstract

Future climate warming in the Arctic will likely increase the vulnerability of soil carbon stocks to microbial decomposition. However, it remains uncertain to what extent decomposition rates will change in a warmer Arctic, because extended soil warming could induce temperature adaptation of bacterial communities. Here we show that experimental warming induces shifts in the temperature–growth relationships of bacterial communities, which is driven by community turnover and is common across a diverse set of 8 (sub) Arctic soils. The optimal growth temperature (*T*_opt_) of the soil bacterial communities increased 0.27 ± 0.039 (SE) and 0.07 ± 0.028°C per °C of warming over a 0–30°C gradient, depending on the sampling moment. We identify a potential role for substrate depletion and time‐lag effects as drivers of temperature adaption in soil bacterial communities, which possibly explain discrepancies between earlier incubation and field studies. The changes in *T*_opt_ were accompanied by species‐level shifts in bacterial community composition, which were mostly soil specific. Despite the clear physiological responses to warming, there was no evidence for a common set of temperature‐responsive bacterial amplicon sequence variants. This implies that community composition data without accompanying physiological measurements may have limited utility for the identification of (potential) temperature adaption of soil bacterial communities in the Arctic. Since bacterial communities in Arctic soils are likely to adapt to increasing soil temperature under future climate change, this adaptation to higher temperature should be implemented in soil organic carbon modeling for accurate predictions of the dynamics of Arctic soil carbon stocks.

## Graphical abstract

Arctic soil bacterial communities incubated at low or high temperatures show divergent community composition and temperature–growth relationships. (Figure was made with Vecteezy.com.)

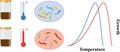

## 1 INTRODUCTION

The functional relationships between temperature and the rate of soil microbial processes, such as growth and respiration, differ markedly between geographic regions in a manner that is largely predictable from climatic averages (Bradford et al., 2019; Dacal et al., 2020; Wang et al., 2021). This correlation between temperature responses and in situ temperature is known as temperature adaptation and can influence the rates of associated biogeochemical rates (Bradford, 2013). Therefore, temperature adaptation of soil microbial processes can influence the vulnerability of soil organic carbon stocks to warming and needs assessment for the prediction of carbon feedbacks to climate change (Allison et al., 2010). For example, microbial respiration in soils from colder regions is generally more responsive to temperature (i.e., higher intrinsic *Q*_10_) than soils from warmer regions (Balser & Wixon, 2009; Dacal et al., 2019; Karhu et al., 2014). Importantly, it is largely unknown whether knowledge about temperature adaptation observed over large‐scale climatic gradients can be used to make predictions about changes to the temperature response of soil bacterial communities at a given locality after exposure to altered temperature regimes. It is particularly important to understand whether extended soil warming can induce temperature adaptation of microbial communities in high‐latitude northern soils, because besides being particularly temperature sensitive, these soils also contain ~50% of the global soil carbon stock (Tarnocai et al., 2009). For this reason, predictions of the fate of global carbon stocks under climate change require mechanistic understanding of the controls of microbial biogeochemistry in Arctic soils (Wieder et al., 2019).

The response of soil microbial activity to temperature can be measured in relation to a variety of biochemical pathways and bio(geo)chemical fluxes (Nottingham, Whitaker, et al., 2019), such as extracellular enzyme activities (Weedon et al., 2014), carbon use efficiencies (Pold et al., 2020; Walker et al., 2018) and respiration (Bradford et al., 2008; Karhu et al., 2014). Many of these measures are potentially confounded by edaphic conditions such as moisture and substrate availability (Davidson & Janssens, 2006; Kirschbaum, 2004; Manzoni et al., 2012). There are therefore advantages in focusing on the temperature response of microbial growth, which can be measured in a way that minimizes the influence of confounding variables (Bååth, 2018; Cruz‐Paredes et al., 2021). The physiological adaptation of bacterial growth to their thermal environment can be described in terms of changes in the parameters of the Ratkowsky model (Bååth, 2018; Ratkowsky et al., 1983), an empirical model for relating microbial growth rates to temperature. By fitting this model to growth rate data, parameters representing the (theoretical) minimal, maximal and optimal temperatures for growth (respectively *T*_min_, *T*_max_ and *T*_opt_) can be estimated and used to compare the temperature response of single strains or, more commonly, communities from different environments (Corkrey et al., 2016). Temperature–growth relationships of bacterial communities estimated in this way have been shown to change along natural gradients of increasing soil temperature following elevation in the Andes (Nottingham, Bååth, et al., 2019), and along a continent‐scale climate gradient in Antarctica (Rinnan et al., 2009). Likewise, 5°C of experimental warming increased the parameters of the microbial temperature–growth relationships in a temperate forest (Rousk et al., 2012). It is therefore likely that long‐term warming causes changes in the temperature–growth relationships of soil bacterial communities, and that as a consequence these communities perform less well at low temperatures (Rinnan et al., 2009). In this study, we define temperature adaptation of soil bacterial communities as a change in aggregate temperature–growth relationship of soil bacterial communities.

Numerous incubation studies have identified the optimal growth temperature (*T*_opt_) as a key parameter for determining how much experimental soil warming is needed to induce temperature adaptation in soil bacterial communities. Specifically, shifts in the overall temperature–growth relationship of a community have been proposed to occur due to selective mortality of microbial taxa when soils are exposed to temperature exceeding the aggregate *T*_opt_ of the microbial community (Bárcenas‐Moreno et al., 2009; Birgander et al., 2013; Donhauser et al., 2020). In the sub‐Arctic, soil bacterial communities typically show a *T*_opt_ between 25 and 30°C and a *T*_min_ of −8 to −6°C (Bååth, 2018; Cruz‐Paredes et al., 2021; Rinnan et al., 2011). Currently, the maximum soil temperatures in the Arctic do not generally exceed 15°C for the majority of the soil habitat (Lembrechts et al., 2021; Table 1) and thus it is unlikely that such a heat‐induced‐death mechanism will play an important role in the (sub‐) Arctic.

**TABLE 1:** Soil characteristics of initial soils

	Site	Total N	Total C	Org. N	Org. C	C:N ratio
1	TH	0.483	11.398	0.483	11.398	23.582
2	TM	0.279	7.564	0.276	7.564	27.106
3	TN	0.492	13.512	0.470	13.017	27.477
4	SA	0.146	2.115	0.138	2.115	14.454
5	SA					
6	FN	0.652	9.934	0.650	9.934	15.242
7	GN	2.218	34.157	2.197	33.743	15.397
8	AB	0.723	47.253	0.723	47.253	65.334

*Note*: Characteristics of sampling sites, including coordinates, mean annual soil temperature, maximum and minimum hourly temperature in °C. Organic nitrogen and carbon content are expressed are depicted the percentage of dry soil weight.

In contrast to the evidence from incubations, a small number of field studies have shown that temperature–growth relationships can also shift with soil temperatures well below the *T*_opt_ of ambient conditions. Shifts in temperature–growth relationships of bacterial communities even occurred when transplanting soil cores to a cooler climate across an ±20°C elevation gradient in the Andes (Nottingham et al., 2021). Moreover, soil bacterial communities altered their temperature–growth relationships at 6–8° degrees of soil warming along a geothermal gradient in Iceland, while the soil temperature of 25°C did not exceed the *T*_opt_ of the bacterial soil communities (30°C) in ambient conditions (Weedon et al., 2022). This implies that, at least in some contexts, long‐term temperature changes can alter temperature–growth relationships of soil bacteria via mechanisms other than heat‐induced mortality under warming scenarios that are realistic for future climate change. However, the empirical basis for this remains limited, and in particular it remains unknown whether the effects observed in Iceland generalize to other cold‐climate soils.

Temperature–growth relationships of soil bacteria might themselves be predictable from information about the taxonomic composition of the bacterial community (Hicks et al., 2021). This is because warming‐induced shifts in temperature–growth relationships have been linked to turnover in community composition (Bárcenas‐Moreno et al., 2009; Donhauser et al., 2020; Weedon et al., 2022). For example, a study of North American forest soils identified 189 bacterial taxa that responded to soil warming, during both heating in a field experiment and in laboratory incubations (Oliverio et al., 2017). To date, a comparable attempt to identify temperature‐responsive species specific to Arctic soils has not been attempted. If found to occur, such a relation between specific (groups of) taxa and physiological responses to warming could provide a tool to predict the response of Arctic soils to changing climate. Specifically, combining community profiling with measures of temperature adaptation could allow for the identification of potential “bioindicator” species specifically related to the temperature adaptation of soil bacterial communities (Bååth, 2018), as has been done previously for salt tolerance (Rath et al., 2018).

Studying warming effects on community composition and physiology is complicated by the fact that soils incubated at a range of temperatures will also differ in substrate availability when sampled after a fixed time interval. As such, direct effects of warming are usually confounded with substrate limitation effects, which can potentially bias the identification of temperature‐responsive bacterial species (Oliverio et al., 2017) and bias estimates of warming effects on physiological processes (Hartley et al., 2008; Karhu et al., 2014; Walker et al., 2018). A common solution is to add excess substrate to temperature incubations to remove indirect effects due to substrate limitation (Dacal et al., 2019), which often alters the soil organic matter quality. As an alternative, the sampling moments can be standardized such that different temperature treatments are compared after a set amount of substrate use or carbon loss (Whittington, 2019).

In this study, we aimed to evaluate whether temperature adaptation by soil bacterial communities occurs when Arctic soils are exposed to experimental warming. Additionally, we asked whether there were general patterns in the temperature–growth relationships and community composition associated with temperature adaptation. To do this, we conducted an incubation experiment with eight soils from four different study sites reaching from the sub‐Arctic to the high Arctic, varying in mean annual soil temperature, vegetation cover and soil type. We tested whether incubation temperatures between 0 and 30°C, reaching >15°C above the observed maximum in situ soil temperatures of all sites, altered temperature–growth relationships and whether the size of the response to incubation temperature is influenced by the MAT of the soils (Donhauser et al., 2020). Second, to separate direct warming effects from indirect effects via substrate depletion, we sampled both after 100 days (T100 samples) and at a variable moment (C15 samples) with timing linked to a set amount of CO_2_ production (as a proxy for substrate use). Lastly, we tested whether eventual shifts in the temperature–growth relationships in response to incubation temperature are related to changes in the bacterial community composition and if we could identify ubiquitous bacterial taxa that respond to soil warming. We hypothesized that (1) the growth parameters of the temperature–growth relationships would increase with higher incubation temperature or when incubation temperature exceeded the *T*_opt_ of the initial bacterial community; (2) and these changes are similar across measurements taken after a fixed incubation period, or fixed amount of cumulative respiration (proxy for substrate availability); (3) changes in temperature–growth relationships are accompanied with a change in the community composition; and (4) there is a set of “bioindicator” taxa common to all soils whose changes in abundance correlate with altered temperature–growth relationships.

## 2 METHODS

### 2.1 Sample collection

To test the generality of our hypotheses across diverse Arctic soil and vegetation types and to identify potential ubiquitous temperature‐responsive bacterial amplicon sequence variants (ASVs), we sampled eight different soils from four locations in the (sub‐) Arctic region in the summer of 2018. The sampling locations varied in mean annual soil temperature at 10 cm depth (from −3.5 to +6.1°C), vegetation cover and soil type (Table 1). We collected two Silandic Andosols from the FORHOT research site in Iceland (64°00′N, 21°11′W), with a dominant cover of *Agrostis capillaris* for the grassland site (GN) and *Picea sitchensis* for the forest site (FN; Sigurdsson et al., 2016). We collected a Histosol from a *Sphagnum* covered bog at the long‐term climate manipulation experiment (Dorrepaal et al., 2004) close to the Abisko Research Station in northern Sweden (AB; 68°21′N, 18°49′E). From the LTER sites at the Toolik Field Station in Alaska, USA (Shaver et al., 2013; 68°38′N, 149°36′W), we collected three soils classified as Typic Aquiturbels. The vegetation cover of LTER Heath (TH) dominated by *Arctostaphylos alpina*, while LTER Moist Acidic Tussock (TM, pH = 3.7) and LTER Non‐Acidic Tussock (TN, pH = 5.9) have *Eriophorum* and *Carex* species as dominant vegetation (Gough et al., 2000; Ping et al., 1998; Street et al., 2007). At Svalbard, we collected two Cryosols from Bjorndalen (78°13′N, 15°19′E). Both sites were characterized by the presence of *Carex* sp., *Salix* sp. and mosses, where the first, SA, showed a dominance of *Carex* and <1 cm organic horizon, and the second site, SB, was mainly covered by mosses and showed a thicker organic horizon, 3–6 cm.

Soil cores were collected with an approximate diameter of 10 cm and depth of 20 cm, and vegetation was removed from the top during sampling. After collection, samples were frozen and shipped cooled to the Vrije Universiteit Amsterdam and stored at −20°C. For further processing, all samples were thawed at 7°C for 7 days, except the Alaskan samples that were thawed at 4°C, to maintain the thawing temperature below the summer soil temperatures. After thawing, the soils were homogenized and passed through a 2 mm sieve. For each soil type, 21 jars were prepared for incubation by adding 20–40 g of fresh weight soil into autoclaved 300 ml mason jars with rubber septa in the lid. The jars and soil were pre‐incubated for 7 days at 7°C. CO_2_ was measured in the headspace after 0 and 7 days using an EGM‐5 infra‐red gas analyzer (PP‐systems) to assess whether soil respiration stabilized over the course of the pre‐incubation. The headspace was then flushed with 0.45 μm filtered air. After the pre‐incubation, 3 replicates of each soil type were placed into incubators set at 0, 5, 10, 15, 20, 25 or 30°C. The CO_2_ concentration in the headspace of the 168 jars was measured at variable intervals between 1 and 7 days depending on the respiration rate and incubation temperature, where the 30°C incubated jars were sampled every day and 0° every 7 days. Once the CO_2_ concentration passed 30.000 ppm, the headspace was flushed with 0.45 μm filtered air. After 15 days, 5 g of samples was taken from the jars at 30°C for soil analysis, DNA microbial community profiling and aggregate temperature relationship measurements. The cumulative amount of CO_2_ produced after 15 days at 30°C was used as reference sampling point, at which all the jars at the other temperatures were sampled between 15 and 285 days (hereafter C15). Additionally, all jars were also sampled after 100 days (hereafter T100). The samples from both time points were used for DNA microbial community profiling and aggregate temperature–growth relationship measurements as described in the following sections.

### 2.2 Characterization of bacterial community composition

Subsamples of 200 mg soil were taken for analysis of soil bacterial community composition. DNA was extracted using a Powersoil Kit (Qiagen), following the manufacturer's protocol, and eluting the final DNA into 60 μl sigma‐sterilized Millipore water. Amplicons were generated by a two‐step PCR of the 16S V4 rRNA gene with primers designed by Caporaso et al. (2011), 515 forward primer (5′‐GTG YCA GCM GCC GCG GTA A‐3′) and 806 reverse primer (5′‐GGA CTA CNV GGG TWT CTA AT‐3′). An initial PCR consisted of an initial denaturation step of 1 min at 98°, followed by 24 cycles of denaturation for 10 s at 98°C, annealing for 30 s at 55°C, elongation for 30 s at 72°C, followed by a final extension of 5 min at 72°C. Amplicons were then 50× diluted in sigma‐sterilized water and subsequently indexed by an 8‐cycle PCR with unique barcode primers for each sample using the same steps as the initial PCR amplification. Purification of the PCR product was done using Ampure XP beads (Beckman Coulter), following the manufacturer's protocol. The amplicons were sequenced over two paired‐end MiSeq Illumina Sequencing runs with V3‐600 cycle chemistry, generating 19,079,107 sequences in total. QIIME 2 was used for processing the resulting sequences (Bolyen et al., 2019). Raw sequences were deposited in the NCBI Sequence Read Archive (BioProject accession number: PRJNA856638). Demultiplexed sequences were truncated at 250 bp for forward and reverse reads. DADA2 (Callahan et al., 2016) was used for dereplication, allowing a maximum expected error of 2. Chimera removal was done internally by DADA2, using the “consensus” mode. ASVs were then aligned using MAFFT (Katoh & Standley, 2013) and phylogenetic distances were calculated using Fasttree (Price et al., 2009). QIIME 2's scikit‐learn naive Bayes machine‐learning classifier (Bokulich et al., 2018) was used for the taxonomic classification based on the SILVA v138 database (Yilmaz et al., 2014). Finally, ASVs matching to mitochondrial or chloroplast sequences were discarded. Lastly, 23 samples were removed from the dataset due to low sequencing depth (<3000 sequences).

### 2.3 Measuring temperature–growth relationships

We used a ^3^H‐leucine growth assay adjusted from Bååth et al. (2001) to estimate the growth rates of soil bacterial communities over a temperature gradient from 0 to 40°C. Each frozen sample was first thawed for 2 days for the recovery of microbial activity (Koponen & Bååth, 2016). Next, 20 ml of sterile deionized water was added to 1 g of soil and vortexed at maximum speed for 2 min. After centrifugation at 1000 *g* for 10 min, 9 aliquots of 1 ml were suspended in 2 ml screw top tubes. For the measurement of leucine incorporation, 20 μl of a mixture of unlabeled leucine and 3H‐leucine (Perkin Elmer) was added to the tubes, resulting in a final concentration of 401 nm and 72.5 kBq ml^−1^. For every sample, one tube was incubated for 20 h at 0°C, 8 h at 4°C, 4 h at 10 and 15°C and 2 h at 24.5, 28.5, 33 and 40°C. As a negative control, for the last tube, 100% TCA was added directly after addition of the leucine mixture to eliminate bacterial growth. To terminate the incubation, 75 μl 100% TCA was added, after which samples were stored for no more than 4 days at 4°C before further processing. The bacterial cells were then washed by centrifugation for 8 min at 1000 *g*, which is then followed by removal of the supernatant and addition of 1.5 ml 5% TCA. The TCA washed samples were then washed in the same manner with 80% ethanol. The final pellets were made by one last step of centrifugation and supernatant disposal, after which 1 m NaOH was added to the pellets, followed by incubation at 90°C for 30–60 min. At room temperature, 1 ml Optiphase Hisafe 3 was added and the tubes were vortexed briefly. Scintillation was measured on a Tricarb 2800T (Perkin Elmer). Leucine incorporation rates were based on the ^3^H‐activity and measured and transformed to nm ^−1^ h^−1^ g dry weight soil using Equation (1):

(1) $$k=\frac{\mathrm{dpm}\times c\;}{t\times w},$$

where *k* is the final leucine incorporation rate (nm ^−1^ h^−1^ g), dpm is the measured disintegrations per minute, *c* the conversion factor from Bq to nm (5.31 × 10^−3^ nm/Bq), *t* is the incubation period in hours and *w* is the added soil dry weight.

### 2.4 Statistical analysis

**FIGURE 1:**
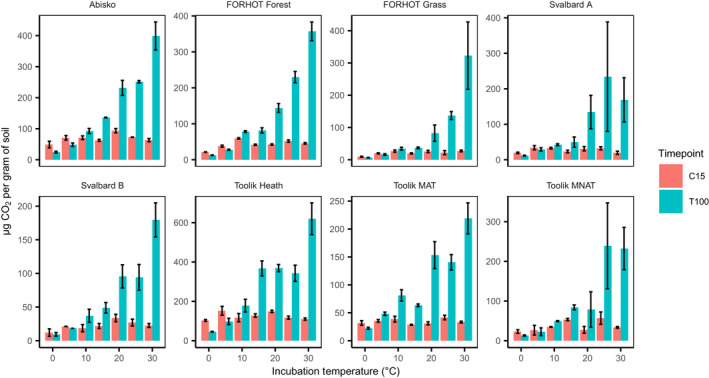
Mean cumulative respiration (μg CO_2_ g^−1^ soil) measured in soils samples from eight Arctic and sub‐Arctic sites incubated between 0 and 30°C (*n =* 3 per site × temperature combination). Cumulative respiration determined at two time points per replicate incubation jar are presented. The blue bars show cumulative respiration after 100 days of incubation (T100). Red bars show cumulative respiration determined at a time point chosen such that an amount of respiration approximately equal to the cumulative respiration after 15 days for samples incubated at 30°C (C15, between 15 and 285 days, depending on soil and temperature). Error bars show standard error of the mean. *Y*‐axis scale varies between panels.

For the calculation of total CO_2_ produced in each jar, we calculated the cumulative respiration after each time interval and linearly interpolated the cumulative respirations between the measurements. Temperature–growth relationships were estimated for each soil sample (i.e., for each of the two sampling points per replicate incubation) by fitting a square root model for bacterial growth (Ratkowsky et al., 1983; Equation 2; Figure 1), to the measured leucine incorporation rates using the *nls.multstart* R‐package (Padfield & Matheson, 2020):

(2) $$\sqrt{\mathrm{Leu}}=a\left(T-T_{\min}\right)\times \left(1-e^{b\left(T-T_{\max}\right)}\right),$$

where Leu is the incorporation rate of leucine, *a* is the slope parameter for growth below the optimum, *T* the temperature of the leucine incorporation assay in °C, *T*_min_ corresponds to the theoretical minimum temperature for growth, *b* is a slope parameter for growth above the optimum and *T*_max_ the theoretical maximum temperature for growth (Figure S1). The temperature of optimal growth, *T*_opt_, was derived numerically, based on the best‐fit parameters.

In previous work, *T*_min_ and the a‐slope parameter were often first estimated by

(3) $$\sqrt{\mathrm{Leu}}=a\left(T-T_{\min}\right),$$

after which these parameters were used as constants in Equation (2) for the second step. In general, only four assay temperatures fitted to the linear section of the Ratkowsky model, limiting the accuracy for fitting Equation (2) in a model. We evaluated both approaches and found negligible differences in estimated parameters from the two approaches, but lower Akaike information criterion (AIC) values when the model in Equation (1) was directly fitted. We discarded 41 samples from the leucine incorporation dataset due to improper storage and/or limited leucine incorporation.

To test the influence of the incubation temperature on the growth parameters, we fitted a mixed‐effect models for the growth parameters *T*_min_, *T*_opt_ and maximum growth rate with incubation temperature and sampling moment (C15 and T100) as fixed effects and soil type and the jar number as random effects. We calculated the marginal *R* ^2^ for the fixed effects of each model using the R‐package *MuMin* (Barton, 2015). To test for potential threshold changes in *T*_min_, we compared linear models and piecewise regression models with *T*_min_ as response variables and the incubation temperature as predictor for each soil type and timepoint combination using the *segmented* R‐package (Muggeo, 2003). We performed a p.score.test on each linear model and evaluated the AIC for both linear and piecewise regression models. To test whether incubation temperature effects on growth parameters were related to the mean annual temperature (MAT) of the sampling sites, we performed an ANCOVA on the relationships between MAT and the estimated coefficient for each site × timepoint combination estimated for the correlation between *T*_opt_ and the incubation temperature.

For the analyses of bacterial community data, the R package *phyloseq* was used, unless stated otherwise. ASVs with proportional abundance lower than 0.001% over the whole dataset were excluded, after which we rarefied our bacterial community composition data to the minimum read count of 3283. We estimated the alpha diversity of the soil bacterial communities using the Shannon index and used mixed‐effects model to test the influence of incubation temperature and sampling moment with soil type and jar as random effects on alpha diversity. Then, we computed the pairwise distance matrix using weighted Unifrac distances (Lozupone & Knight, 2005). To study the drivers of community composition, we performed a series permutational multivariate analysis of variance (PERMANOVA; Anderson, 2001) tests on the calculated dissimilarity distances to determine the influence of incubation temperature, sampling moment (C15 or T100) and soil type on bacterial community composition. We also performed a PERMANOVA test on the calculated dissimilarity distances at C15, with incubation temperature and cumulative respiration amount as predictor variables. Additionally, we tested for a correlation between in situ *T*_opt_ and the overall soil bacterial community composition with PERMANOVA. Last, we performed a PERMOVA test on the Unifrac distances of each soil separately at C15 to test whether the bacterial community composition responded to incubation temperature.

To detect the possible abundance differences of ASVs along the incubation temperature gradient, we performed a differential abundance analysis for each soil type and sampling moment individually at phylum, family and ASV levels using the ANCOMBC 1.1.4 package (Lin & Peddada, 2020). In this analysis, we tested whether the differential abundance of each taxon was correlated to the incubation temperature, using Bonferroni method for *p*‐value adjustment for multiple comparisons and default settings of the package. ASVs were considered “true” temperature responders when the ASVs responded positively/negatively to the incubation temperature at C15 for at least two soils. The program R (v4.0.2) was used for all statistical analyses (R Core Team, 2020). Data for CO_2_ measurements and parameters of the temperature–growth relationships are available via Figshare (https://doi.org/10.6084/m9.figshare.19516777 and https://doi.org/10.6084/m9.figshare.19516780).

## 3 RESULTS

### 3.1 Incubation temperature and soil type influence soil respiration rates

Respiration rates increased with incubation temperature and differed between the 8 soil types, ranging between 0.25 and 2 μg^−1^ g^−1^ day at 10°C (mixed‐effects model; marginal *R* ^2^ = .87, *p* < .001). The cumulative amount of CO_2_ produced after 100 days varied by a factor of 14–69 between soils incubated at 0 and 30°C depending on the soil type (mixed‐effects model; marginal *R* ^2^ = .86, *p* < .001, Figure 1). At C15, differences between minimum and maximum cumulative respiration were reduced to 1.44‐fold to 2.99‐fold across the soil types. There was a weak correlation between incubation temperature and CO_2_ produced at C15 (mixed‐effects model; marginal *R* ^2^ = .01, *p* < .01, Figure 1), but when the soils at 0°C were excluded, it was no longer significant (*p* = .30).

### 3.2 Linear change in temperature–growth relationships with incubation temperature

**FIGURE 2:**
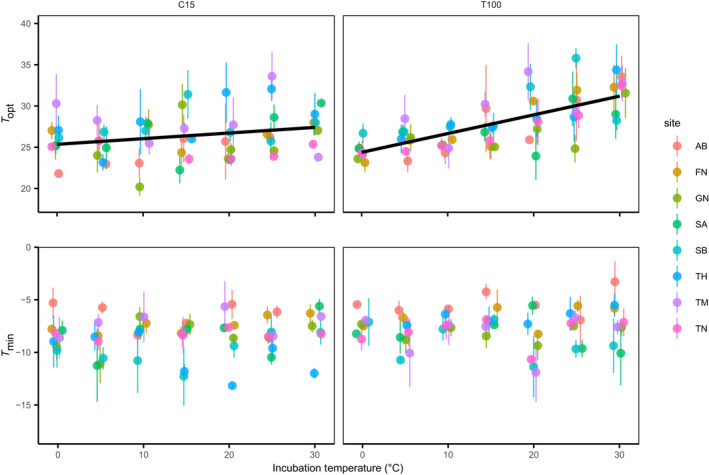
Relationships between estimated parameters of the temperature–growth function (upper panels: Theoretical minimum temperature for growth, *T*_min_; lower panels: Estimated optimal growth temperature, *T*_opt_) and soil incubation temperature. Parameters were estimated with leucine incorporation assay using 9 temperatures between 0.5 and 40°C. Assays were performed using soil samples taken at both C15 (left panels) and T100 (right panels, see methods). Points and error bars show means ± standard error per site × timepoint combination (*n* = 3). Points are jittered horizontally for legibility. Lines represent significant linear regressions (*p* < .05).

The temperature–growth relationships of the bacterial communities were influenced by the incubation temperature. *T*_opt_ showed a linear increase with the incubation temperature (mixed‐effects models; *p* < .05, Figure 2), as piecewise regression showed higher AIC scores than the mixed‐effect linear models. The increase in *T*_opt_ was significantly higher in T100 samples compared to C15 samples (*p* < .001), increasing by 0.27°C ± 0.039 (SE) and 0.07 ± 0.028°C per °C of incubation temperature, respectively. The magnitude of the incubation temperature effect on *T*_opt_, was not significantly related to the MAT of the sampling site (ANCOVA of *T*_opt_ slopes per site, *p* > .05, Figure S6). Alongside the *T*_opt_ effects, there was a weak but significant positive relationship between *T*_max_ and the incubation temperature (mixed‐effects models; *R* ^2^ = .08, *p* < .05), with no significant difference in *T*_max_ between the two sampling moments (*p* = .12). Lastly, *T*_min_ of the incubated soil bacterial communities was not significantly influenced by the incubation temperature in either a linear mixed model (*p* = .47, Figure 2) nor in piecewise regression models (*p* > .05 for all models). In total, the temperature range of growth (*T*_max_ − *T*_min_) increased 0.14°C per °C of incubation temperature (*p* < .01) at T100, but at C15 the temperature range of growth was not significantly different across the incubation gradient. The growth rates of the bacterial communities, here defined by the maximum leucine incorporation rates at *T*_opt_, showed no significant correlation with the incubation temperature at C15. However, microbial growth rates at T100 significantly decreased by 36.2% from 0 to 30°C (mixed‐effect models; marginal *R* ^2^ = .07, *p* < .001). Overall, the temperature and sampling moment influenced the *T*_opt_ and *T*_max_ parameter regardless of the sampling site MAT, while *T*_min_ was not significantly altered, resulting in a broader temperature range of growth (Table 2).

**TABLE 2:** Regression results of temperature–growth relationships parameters

	*T*_opt_		*T*_min_		*T*_max_	
	Estimate	*t* value	Estimate	*t* value	Estimate	*t* value
Intercept	25.3	40.6***	−8.7	−18.0***	43.7	−18.0***
Temperature	0.1	2.5*	0.0	1.3	0.1	1.3
Sampling moment	−0.9	−1.3	0.9	2.0	−0.9	2.0
Temperature × sampling moment	0.2	3.9***	0.0	−0.5	0.0	−0.5

*Note*: Fixed effects from mixed‐effects models relating estimated for each growth parameters to incubation temperature parameter, *T*_min_ and *T*_max_, with site and replicate were included and sampling moment (C15 and T100) as fixed effects and soil type and the jar number as random effects. Asterisks denote significance level (* = *p* <0.05, *** = *p* < .001).

### 3.3 Incubation temperature influences bacterial community composition at both sampling moments

**FIGURE 3:**
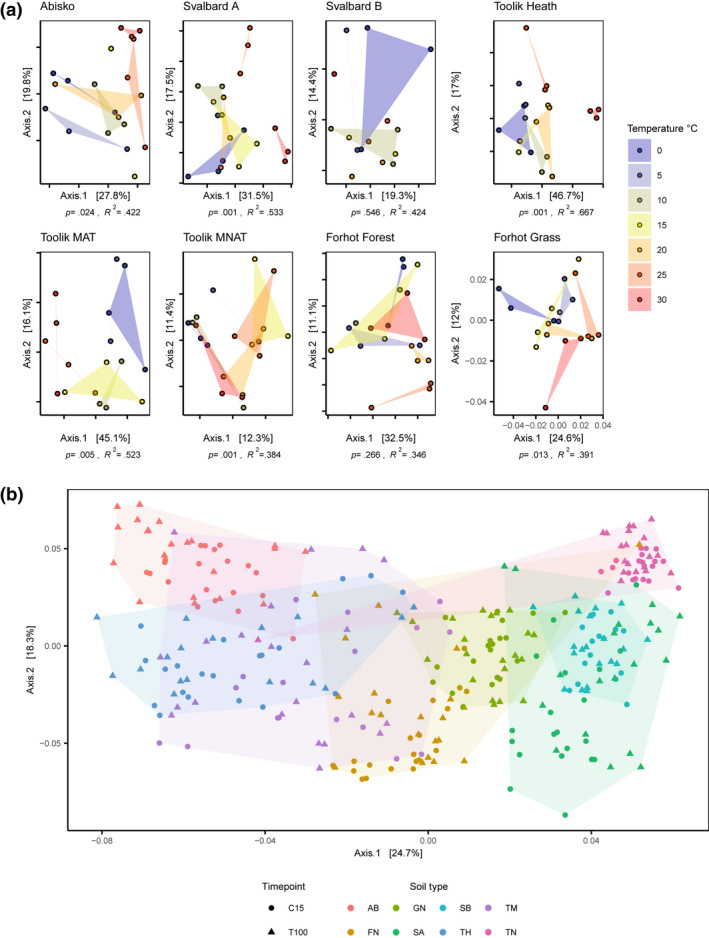
Bacterial community dynamics along the incubation temperature gradient. (a) Principal coordinate analysis using weighted Unifrac distances computed from soil bacterial community profiles generated from 16S amplicon sequencing. Samples taken at C15 for each individual soil type are shown. Blue to red points and convex hulls indicate incubation temperature (0–30°C, *n* = 1–3 for each site × temperature combination). (b) Principal coordinate analysis using weighted Unifrac distances computed from 16S amplicon profiles for all soil, incubation temperature and time points. The majority of variance is related to the soil type (indicated by the hull color).

Overall, 5836 ASVs were observed with a mean abundance above 0.001%, of which none were present in all of the eight soil types. Proteobacteria (29%), Acidobacteriota (22%), Actinobacteriota (15%), Bacteroidota (10%) and Verrucomicrobiota (7%) were the most abundant phyla across all soil samples. The soil type explained the largest part of the variation in bacterial community composition (PERMANOVA *R* ^2^ = .54, *p* < .001, Figure 3b), while both the incubation temperature (*R* ^2^ = .04, *p* < .001) and the sampling moment (*R* ^2^ = .01, *p* < .001) had a significant effect on the bacterial community composition, as well as the interactions between all variables (Table 3). Despite significant differences in the amount of soil respiration across the temperature gradient at the C15 sampling moment, the soil bacterial community composition of all samples was not significantly related with the respiration amount at C15 (PERMANOVA; *R* ^2^ = .004, *p* = .131). At C15, 6 out of 8 soil types showed a significant change in soil bacterial community composition with incubation temperature in a soil‐specific PERMANOVA (*R* ^2^ > .3, *p* < .05, Figure 3a). The Shannon index of the soil bacterial communities did not correlate significantly with incubation temperature (mixed‐effects model, *p* = .76).

**TABLE 3:** Results of permutational multivariate analysis of variance on bacterial community composition

	df	SumsOfSqs	MeanSqs	F.Model	*R* ^2^	Pr.F.
Soil	7	1.164	.166	73.584	.543	0.001
Temperature	6	0.088	.015	6.460	.041	0.001
Timepoint	1	0.016	.016	6.896	.007	0.001
Soil × temperature	42	0.208	.005	2.196	.097	0.001
Soil × timepoint	7	0.044	.006	2.774	.020	0.001
Temperature × timepoint	6	0.032	.005	2.393	.015	0.001
Soil × temperature × timepoint	40	0.142	.004	1.575	.066	0.001
Residuals	199	0.450	.002		.210	
Total	308	2.144			1	

*Note*: Effects of the soil type, temperature and sampling moments on the bacterial community composition. Results of permutational multivariate analysis of variance model on the weighted UniFrac distances for all the soil bacterial community observations. Samples were compared composition based on weighted Unifrac distances derived from 16S rRNA gene amplicon sequencing profiles.

### 3.4 Lack of common temperature‐responsive species

**FIGURE 4:**
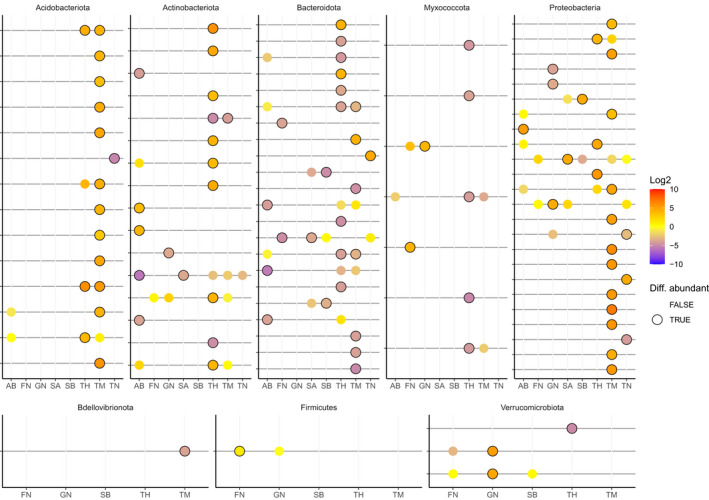
Differential abundance analysis of bacterial amplicon sequence variants (ASVs), shown in log2 fold difference per °C incubation temperature (color of points). Results are shown for C15 samples. Only ASVs significantly responding in at least one soil type are shown. For each ASV × soil combination: Points with dark borders indicate significance (ANCOM‐BC *p* < .05, Bonferroni false discovery rate correction), points without borders are present but not significant, absence of points indicates ASV was not detected.

While there were significant changes along the incubation gradient in overall bacterial community composition, no clear taxonomic patterns in abundance were shown across the soils at either phylum or family level. Differential abundance analysis on ASV count data identified 240 ASVs at T100 and 111 AVSs at C15 that changed significantly in abundance along the incubation temperature gradient in at least one of the soil types (Figure 4). In all, 19 of these ASVs showed significant differential abundance across the temperature gradient for both timepoints in at least one soil type. Only seven of the ASVs (belonging to the orders Micrococcales, Acidobacteriales, Xanthomonadales and Sphingobacteriales) responded to temperature in two or more soil types at C15 (Table 4).

**TABLE 4:** Temperature‐responsive bacterial ASVs

ASV	Order	Family	Genus	Species	Observed in soils (*n*)	Soils diff. abundant (*n*)	Soils
1	Micrococcales	Microbacteriaceae	Unknown	Unknown	6	3	AB, TM, SA
2	Acidobacteriales	Acidobacteriaceae_(Subgroup_1)	Unknown	Unknown	2	2	TH, TM
3	Sphingobacteriales	Sphingobacteriaceae	Mucilaginibacter	Unknown	3	2	TH, TM
4	Sphingobacteriales	env.OPS_17	env.OPS_17	uncultured_bacterium	5	2	FN, SA
5	Acidobacteriales	Acidobacteriaceae_(Subgroup_1)	Occallatibacter	uncultured_Acidobacterium	2	2	TH, TM
6	Xanthomonadales	Rhodanobacteraceae	Rhodanobacter	uncultured_prokaryote	3	2	TH, TM
7	Sphingobacteriales	Sphingobacteriaceae	Mucilaginibacter	Unknown	3	2	TH, TM
8	Acidobacteriales	Acidobacteriaceae_(Subgroup_1)	Occallatibacter	uncultured_Acidobacterium	2	2	TH, TM
9	Micrococcales	Micrococcaceae	Unknown	Unknown	3	2	FN, GN

*Note*: Taxonomic assignments of the temperature‐responsive amplicon sequence variants (ASVs) present in two or more soil types.

## 4 DISCUSSION

In this study, we examined the effect of incubation temperature on the temperature–growth relationships and community composition of Arctic soil bacterial communities. We provide evidence for temperature adaptation of the soil bacterial communities on the scale of weeks to months, as the community‐aggregated optimal temperature for growth (*T*_opt_) shifted in response to increasing incubation temperature at both sampling moments. Notably, there was no corresponding change in *T*_min_ for the same samples. These changes were presumably caused by a turnover in the community composition towards increased abundance of warm‐adapted species since the soil bacterial community composition changed along the temperature gradient at both sampling moments for most soil types.

### 4.1 Temperature–growth relationships show community adaptation to warmer temperatures

The observation that temperature–growth relationships changed all along the incubation temperature gradient contradicts part of our first hypothesis that such a shift would only occur at temperatures above the initial *T*_opt_. It has been previously proposed that bacterial species die due to heat stress at temperatures above the *T*_opt_ of the soil bacterial community (Bárcenas‐Moreno et al., 2009), which has been associated with an increase in maximum growth rates and a decline in species richness in short‐term incubation studies (Donhauser et al., 2020). In this present study, four of the eight soil types (FN, SA, SB and TN) were exposed to temperatures more than 5°C above their corresponding initial *T*_opt_ and did not show a corresponding abrupt change in either *T*_opt_ or *T*_min_. The observed stability of *T*_min_ contradicts the findings of the majority of earlier incubation studies, which raises the question whether our result is attributable to technical and/or experimental design issues. An additional simulation power analysis showed that our study design was adequate for detecting the expected changes in *T*_min_ (Supporting Information). Moreover, the lack of an observation of increased maximum growth rates or decreasing species richness of the bacterial communities suggests that the “heat‐induced death” mechanism was unlikely to be causing the observed patterns.

The non‐effect of incubation temperature on *T*_min_ meant that, in our experiment, bacterial soil communities effectively broadened their temperature range of growth, through increases in *T*_opt_ and *T*_max_. While previous work has proposed that the interval between *T*_max_ and *T*_min_ is stable (Bååth, 2018; Li & Dickie, 1987), variation of temperature ranges of growth has been observed in response to soil temperature fluctuations in at least some cases (van Gestel et al., 2013). We observed a broader range between *T*_min_ and *T*_max_ with increasing incubation temperatures. Due to the difficulties of reliably measuring *T*_opt_ and *T*_max_ (Rinnan et al., 2009), not all previous studies have reported the full set of growth parameters, which complicates comparison (Birgander et al., 2013; Rinnan et al., 2009). However, it has been shown that alpine soil bacterial communities varying in *T*_opt_ 27.3–30.3°C also increased in *T*_opt_ without changing *T*_min_ when incubated at 25°C, while at 35°C both *T*_min_ and *T*_opt_ changed (Donhauser et al., 2020). Recent work shows that Antarctic bacterial communities varied little in their range from *T*_min_ to *T*_opt_ between fluctuating thermal regimes in soil and stable maritime thermal regimes (van Gestel et al., 2020). More research is needed to develop a framework for understanding how the temperature range of growth is affected by environmental temperatures. In turn, such a framework could be important for understanding the mechanisms that lead to temperature adaptation of soil bacterial communities under different warming scenarios.

We hypothesized that soils from colder environments would respond more strongly to the incubation temperature gradient as these soils were exposed to temperatures further above the in situ thermal regime. However, despite the *T*_opt_ of in situ communities ranging from 22.5 to 31.5°C across the eight soils, there was a common slope of the response of the same parameter to the incubation temperature. In other words, the magnitude of the response of *T*_opt_ of Arctic soil bacterial communities to the incubation temperature was not related to the in situ soil temperature regime, and therefore resulted in *T*_opt_ varying between soils at the same incubation temperature. On the one hand, this implies that all soils contain bacteria with a sufficient range of temperature traits to allow comparable responses to warming (Wang et al., 2021). On the other hand, it is possible that the shifts in *T*_opt_ of the soil communities require time scales greater than the length of this study, and that given sufficient time to equilibrate, all soils would eventually attain a temperature‐specific *T*_opt_ (Nottingham et al., 2021). Therefore, more work on field warming experiments could help to define the roles of long‐term dynamics and legacy effects on the adaptability of soil bacterial communities to soil warming.

The lack of threshold dynamics in the composition of the soil bacterial communities across the temperature gradient (Figure 3) indicates that the shifts in temperature–growth relationships were caused by a gradual turnover in the bacterial community. Such a pattern could emerge from species sorting due to environmental filtering (Leibold et al., 2004). Importantly, we observed that these compositional changes occurred in C15 samples, when differences in substrate availability between temperature treatments were minimized, indicating that direct temperature effects were at least partially responsible. While it is possible that temperature–growth relationships can change without compositional shifts, for example, through genotypic (Chase et al., 2021) or phenotypic adaptation, these mechanisms have been considered less plausible in incubation experiments because of the insufficient duration for evolutionary processes, and because of the minor changes in temperature response typically associated with phenotypic changes (Bárcenas‐Moreno et al., 2009). Our findings provide further evidence that community turnover is causing the shifts in temperature–growth relationships, as has been previously been found in both incubation and field studies (Donhauser et al., 2020; Weedon et al., 2022).

**FIGURE 5:**
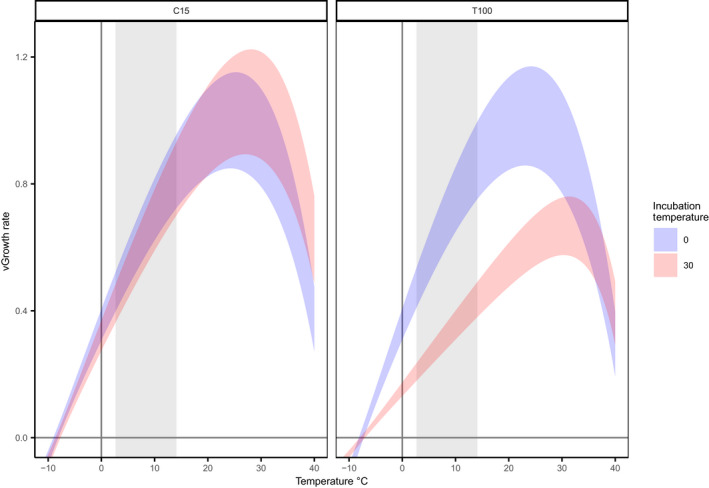
Estimated effect of soil incubation temperature on temperature–growth relationships of bacterial communities. Curves represent predicted responses of short‐term growth rates to temperature between −10 and 40°C. Separate curves are calculated for each sampling moment (left and right panels) and for soils incubated at 0 and 30°C (blue and red curves) using the parameter estimates for the Abisko soils. Bands show 90% confidence intervals estimated by bootstrapping fixed effects of incubation temperature and sampling moment from the mixed‐effect models for each of the 4 combinations of growth parameters (*n* = 1000). Grey areas show the range of average daily summer soil temperatures over the 8 sites used in the present study.

Our comparison of C15 and T100 samples provides insight into drivers of the shifts in the temperature–growth relationships of soil bacterial communities under future warming. As for community composition, the changes in temperature–growth relationships appear to be at least partly driven by direct temperature effects, since incubation temperature effects on temperature–growth relationships were significant at C15. Nevertheless, the effect of incubation temperature on temperature–growth relationships was larger at T100 than C15, which refutes our second hypothesis. The sampling moment after 100 days was substantially longer than previous studies, in which incubation periods of between 72 h and 1–2 months are typical (Birgander et al., 2013; Donhauser et al., 2020; Ranneklev & Bååth, 2001). Such a longer incubation presumably allows for a greater number of generations within the bacterial community and consequently a greater degree of community turnover (compared to C15 samples) in the warmer treatments. On the other hand, limiting substrate availability could possibly drive the competitive benefits of bacterial species performing well at higher temperatures under resource limitations. In turn, this could lead to a larger effect of incubation temperature on the aggregate temperate growth relationships of soil bacterial communities. Overall, our results suggest that substrate limitation and/or a larger number of generations are most likely responsible for the more pronounced temperature effects in the warmer treatments after 100 days. We are unable to test which of these mechanisms predominates; however, the temperature‐related decrease of maximum growth rates in T100 samples suggests that substrate limitation is playing a role (Figure 5).

The longer incubation period and separation of direct and indirect temperature effects in this study also allow us to make connections between observations from incubation and field studies. Long‐term warming studies under field conditions (>4 years) have shown that temperature–growth relationships can change without exceeding the *T*_opt_ of the initial soil microbial community (Nottingham et al., 2021; Weedon et al., 2022). Our study shows that this effect is also reproducible under incubation conditions and that this could be partially driven by substrate limitation and exposure time under warmed conditions. Indeed, the temperature adaptation of soil bacterial communities to temperature below *T*_opt_ can be a long‐term process, for example, taking as long as 11 years after transplanting soil to a cooler climate (Nottingham et al., 2021). This indicates that, depending on the magnitude of the temperature change and new climate, temperature adaptation induced by soil warming might take more generations than previously assumed. Altogether, this shows that bacterial communities can adapt to relatively modest increases in temperature after extensive exposure. However, previous field studies with warming by open top chambers showed no significant change in temperature under moderate warming of 1–2°C, which could be due to measurement error larger than the expected effect size (Rinnan et al., 2009, 2011). This study shows that more research is needed to evaluate the drivers of temperature adaptation by soil bacterial communities and whether temperature adaptation will occur under moderate soil warming in field conditions.

### 4.2 Compositional changes in response to warming: Are there bioindicators?

The simultaneous shifts in temperature–growth relationships and community composition under warmed conditions in this study support our third hypothesis (Donhauser et al., 2020; Weedon et al., 2022). Additionally, 4 of 8 soils showed strong similarity in the response to temperature in terms of community dynamics (Supporting Information). Since the incubation temperature could be an environmental filter for community assembly (Leibold et al., 2004), these shifts could indicate that the community turnover led to a dominance of warm‐adapted species. It is likely that temperature‐responsive taxa can only be identified on a species or ASV level, as traits related to the thermal performance of microbial species are hypothesized to be phylogenetically conserved at a relatively shallow level (Martiny et al., 2015). In accordance with this, we found no universal temperature response when community data were analyzed according to bacterial phyla. We therefore used differential abundance analysis to identify which species were responsive to the incubation temperature at limited substrate effects (C15). We detected 111 ASVs at C15 as differentially abundant across the temperature gradient in at least one soil type, of which 69 ASVs were also differentially abundant at T100. Isolation of these temperature‐responsive ASVs will be needed to verify whether the ASVs are characterized by higher optimal growth temperatures than other community members. Previously, it has been proposed that temperature‐responsive species in the bacterial community could be used as indicators for the estimation of community‐wide temperature–growth relationships (Hicks et al., 2021). However, in this study, only few ASVs were differentially abundant in more than one soil type. At C15, seven ASVs responded in two or more soil types (Table 4). The soil specificity of temperature‐responsive species suggests a limited utility of such “bio‐indicators” across soils. Indeed, in a biogeographic study, only 15 (operational taxonomic units at 97% similarity) were ubiquitously present across the 43 Arctic sampling sites (Malard et al., 2019). This high biogeographic heterogeneity combined with the aforementioned limited taxonomic signal of temperature‐responsive species implies that bio‐indicator species might not be suited for all soils. Therefore, the determination of temperature–growth relationships of soil bacterial communities is likely to be more efficient through direct measurements such as the leucine assays employed in our study (Hicks et al., 2021).

### 4.3 Implications and conclusions

Our findings imply that temperature–growth relationships of Arctic soil bacterial communities will change under warmed conditions. However, we show that the extent to which this occurs under field conditions will be determined by the degree of temperature adaptation, and the range of temperatures that soils are subject to under warming, as well as the dynamics of substrate availability. For example, based on our data, at C15 there are minimal differences in the predicted growth rates of the cold and warm exposed bacterial communities between 0 and 20°C (Figure 5), which represents a scenario where substrate supply is comparable to the pre‐warming state. However, if adaptation in the field is closer to the scenario related to T100 measurements, because of longer exposure and potential substrate limitation, this could induce substantial change in the growth rates at temperatures relevant to near‐term warming in Arctic soils. These results indicate that separation of these drivers will be important for understanding the potential for changes in growth rates of Arctic soil bacterial communities under future climate conditions.

As follow‐up to this study will be important to assess whether other soil decomposer communities respond to soil warming as well. Fungal communities respond to temperature in similar way to soil bacterial communities (Bárcenas‐Moreno et al., 2009; Birgander et al., 2018; Nottingham, Bååth, et al., 2019; Pietikäinen et al., 2005), but so far no studies have shown shifts in the temperature–growth relationships of soil fungal communities (Birgander et al., 2018). It will also be important to assess the influence of shifts in microbial temperature–growth relationships on a broader range of soil biogeochemical processes, such as soil respiration and nutrient cycling. So far, studies have shown that implementation of microbial processes, such as altering the temperature sensitivity of the enzymatic parameters (Wieder et al., 2013) and/or the carbon use efficiency of soil bacterial communities (Allison et al., 2010; Palacios et al., 2021; Wieder et al., 2013), substantially influences model projections of soil organic carbon (SOC) stock under warmed conditions. However, shifts in temperature–growth relationships have been shown to have only limited effects on soil respiration in a temperate forest soil, as the increased growth reduced under warmed conditions due to possible reduction in substrate availability (Rousk et al., 2012). Currently, less is known about the balance of warming and substrate‐mediated feedbacks in Arctic organic‐matter‐rich soils. Microbial growth rates in microbial‐explicit SOC models are often defined by the uptake rate of substrates into the microbial biomass. Making this uptake rate temperature dependent, following the Ratkowsky equation for bacterial growth, would appropriately represent temperature–growth relationships in SOC models. These models could then be used to identify where changing temperature–growth relationships will have large implications for SOC stocks on the global scale.

The temperature adaptation of Arctic soil bacterial communities, here shown under experimental warming, indicates that the microbial response to global warming could influence carbon cycling in Arctic terrestrial region. However, it remains a challenge to link the temperature adaptation to specific species that can be used as bioindicators for understanding and predicting the functional implications of temperature adaptation of soil communities. Furthermore, we propose incorporating shifts in temperature–growth relationships into earth‐system models to assess the potential magnitudes of their impacts on soil carbon cycling.

## CONFLICT OF INTEREST

The authors declare no conflict of interest.

## Supporting information


Figure S1.

Figure S2.

Figure S3.

Figure S4.

Appendix S1.

## Data Availability

The data that support the findings of this study are available on Figshare (https://doi.org/10.6084/m9.figshare.19516780 and https://doi.org/10.6084/m9.figshare.19516777) and the NCBI Sequence Read Archive under BioProject number PRJNA856638.
