# Supplementary figures and images for: Optimal growth temperature of Arctic soil bacterial communities increases under experimental warming
Source: Glob Chang Biol. 2022 Jul 24;28(20):6050–64. doi: 10.1111/gcb.16342 (PMC9546092; doi:10.1111/gcb.16342)

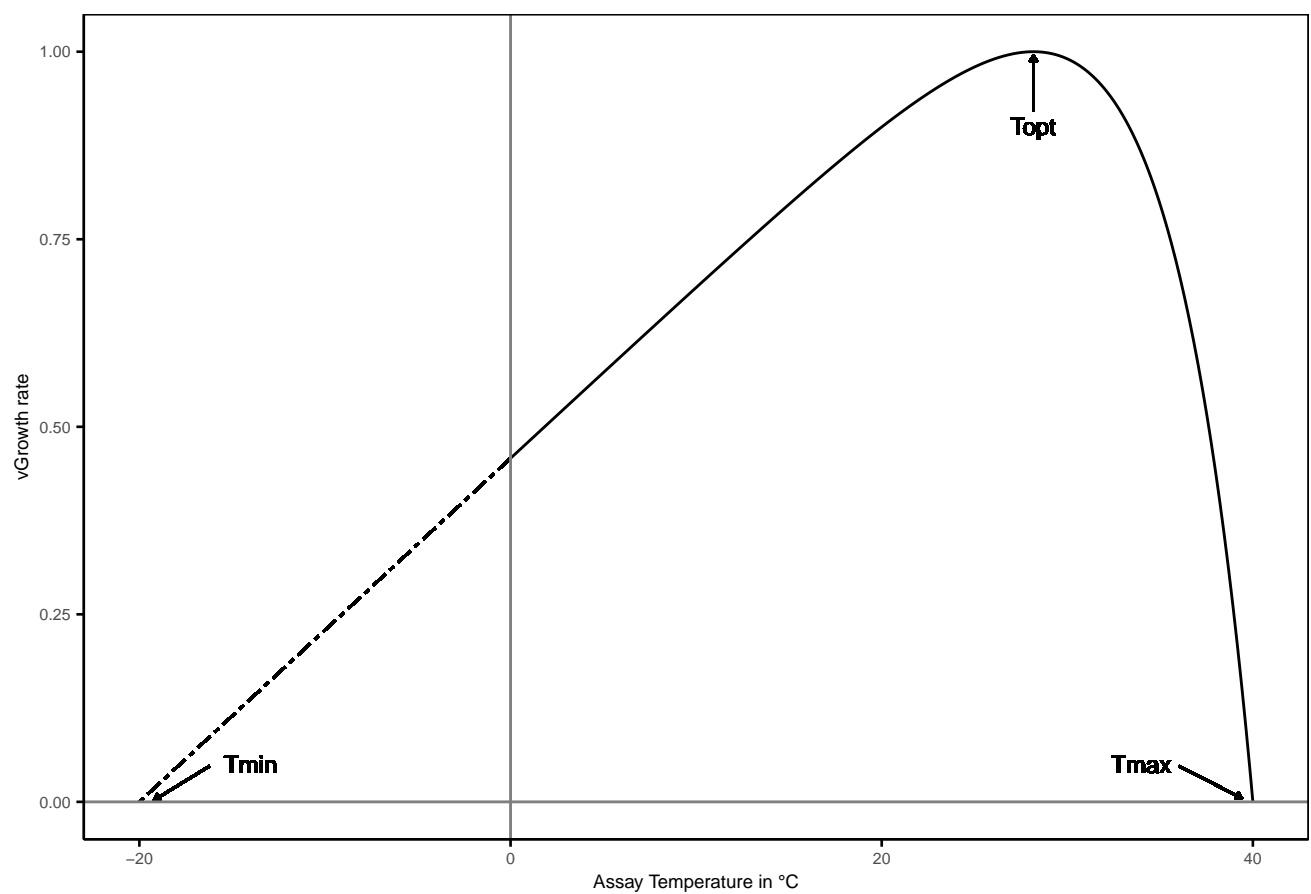

Supplement: Supplementary file 1 — Figure S1. [file GCB-28-6050-s001.pdf]

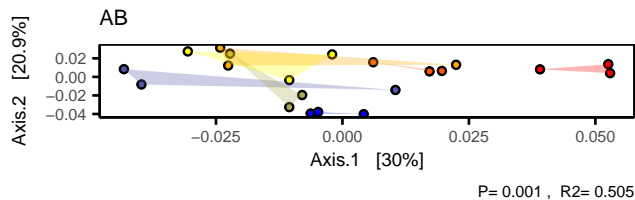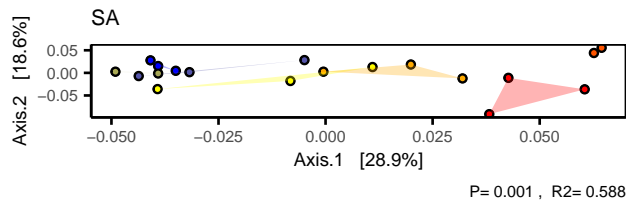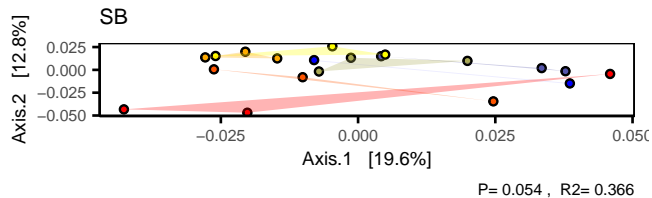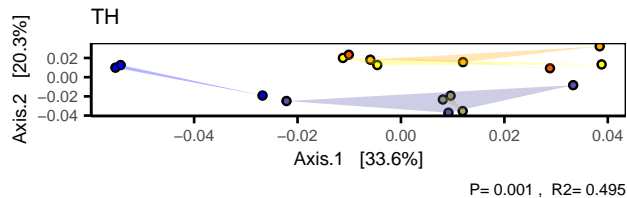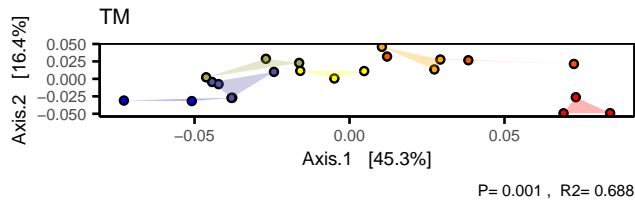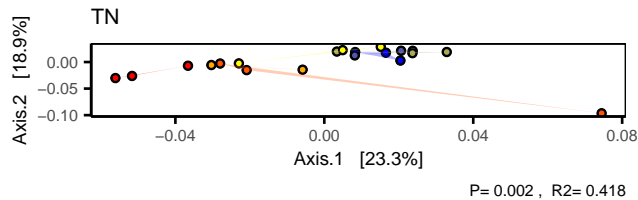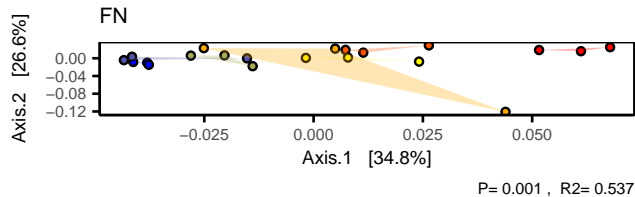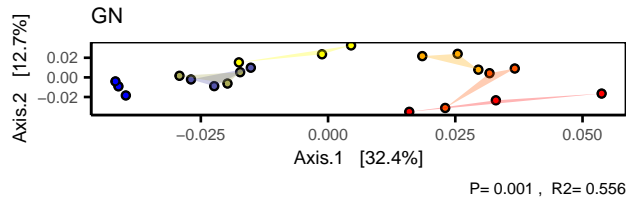

Supplement: Supplementary file 2 — Figure S2. [file GCB-28-6050-s005.pdf]

Bacterial community composition  
at T100

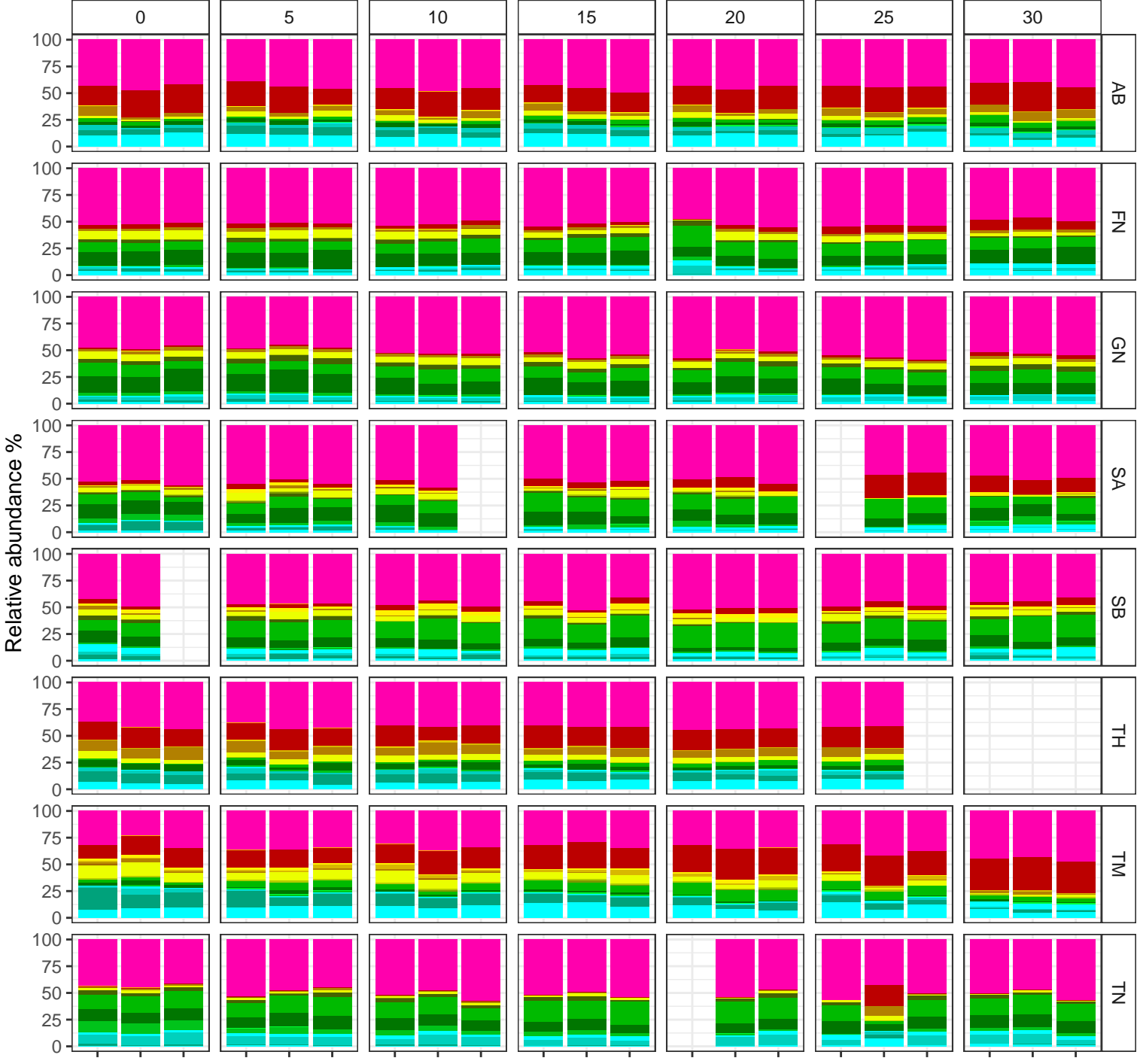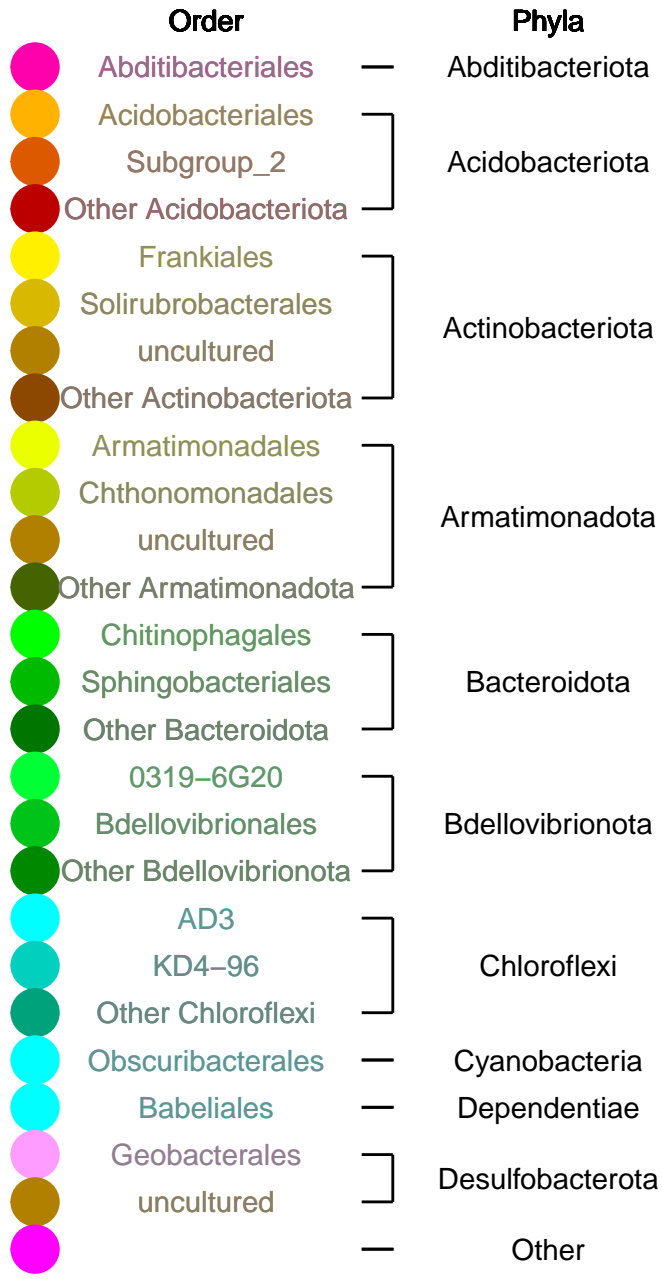

Supplement: Supplementary file 3 — Figure S3. [file GCB-28-6050-s002.pdf]

Bacterial community composition  
at C15

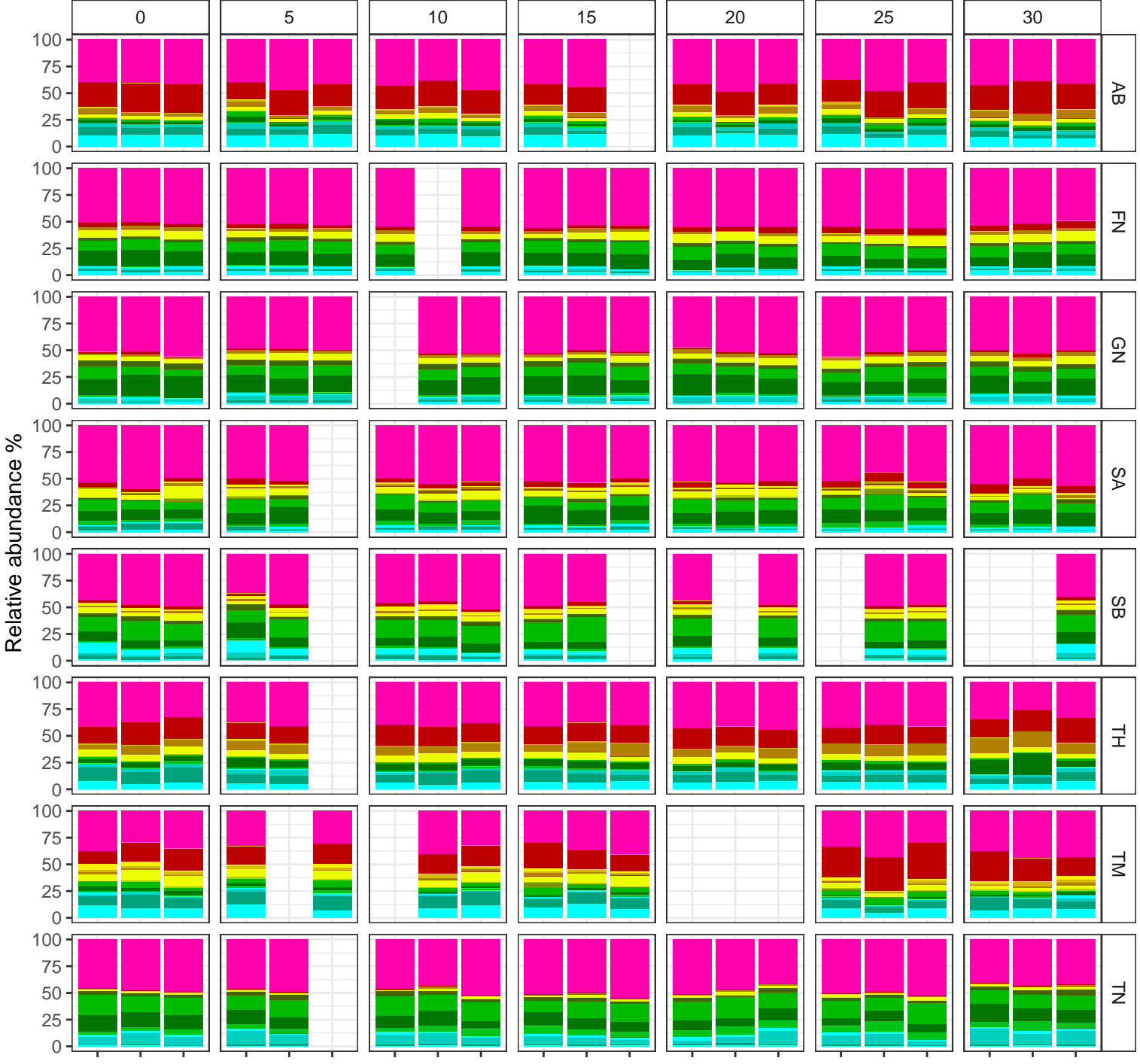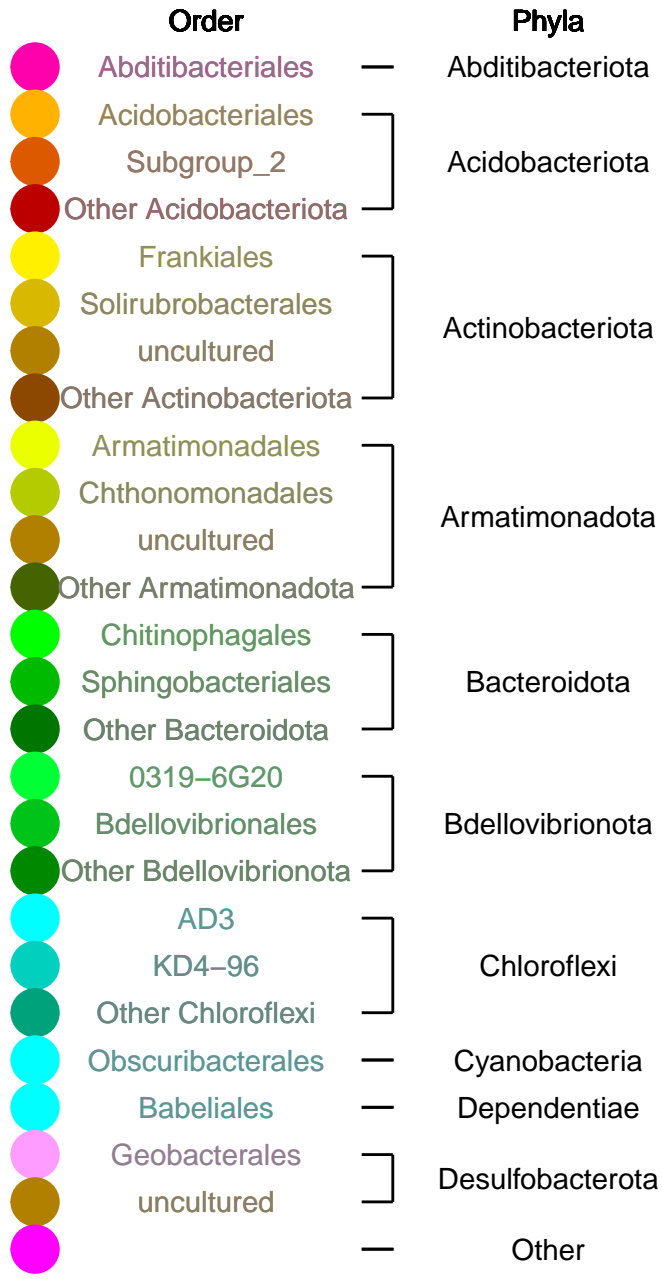

Supplement: Supplementary file 4 — Figure S4. [file GCB-28-6050-s003.pdf]
