# Supplementary material for: Optimal growth temperature of Arctic soil bacterial communities increases under experimental warming
Source: Glob Chang Biol. 2022 Jul 24;28(20):6050–64. doi: 10.1111/gcb.16342 (PMC9546092; doi:10.1111/gcb.16342)
Supplement: Supplementary file 5 — Appendix S1. [file GCB-28-6050-s004.docx]

Supplementary Methods

# Power analysis on Tmin measurements

We performed a simulation power analysis (Arnold et al., 2011) to evaluate the possibility that the observed null result was due to the relatively large sample variance of Tmin (mean CV = 14.9%) and modest amount of replication (n = 3 per soil and incubation temperature combination).Taking a Tmin increase of 0.8 °C per °C incubation temperature above the initial Topt reported by Birgander et al., (2018) as a starting point, we assumed an effect size of 4°C difference in Tmin between soils incubated > 5°C above and below the initial Topt. Simulations with alpha set at 0.05 showed that 80% power would already be expected above for a difference of Tmin of 1.5°C between samples incubated above or below (Figure 1).


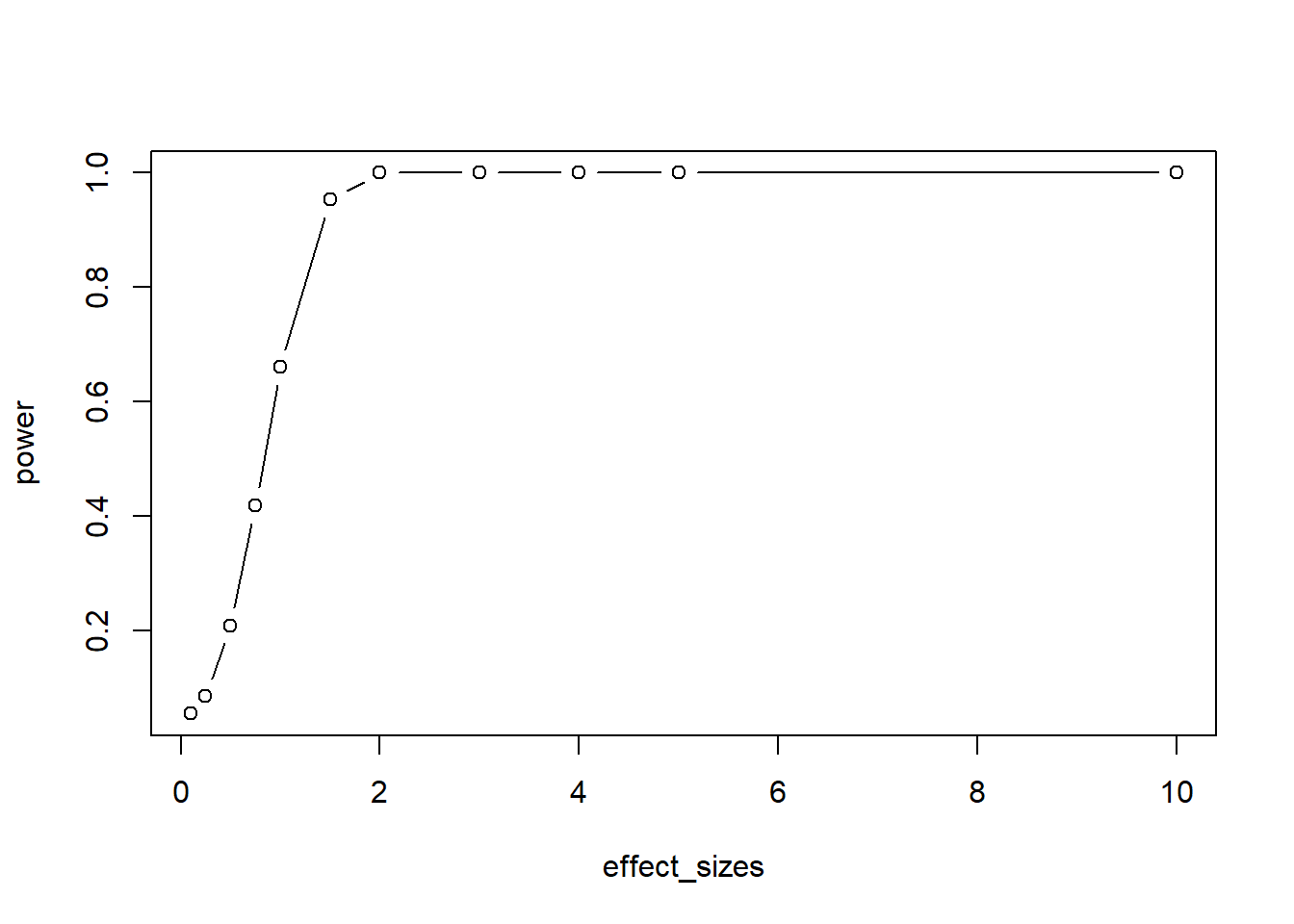


**Figure 1**. Estimated power for effect size from 0 to 5 for change in Tmin above and below the initial Topt of incubated soils.

# Comparison of community responses between soil types

The differential abundance analysis presented in the main text compares the response of bacterial communities to incubation temperature in terms of shared responsive species. At a more general level, it is possible to test the similarity or divergence of community composition response to temperature. We do this by performing Mantel tests between the distance matrices of all 8 soil types to determine the correlation between community response to temperature between the soils. We converted the resulting correlation R^2^ values to distances, √(2 (1-R^2^)) and performed hierarchical cluster analysis on the distance matrix with ‘complete linkage’ method. From this we conclude that at least four of the sites showed highly similar community-level responses to incubation temperature (Table 2; P<0.05, R2 > 0.6). The remaining four were either not affected by incubation temperature or showed correlation in community response.

**Supplementary Table 1. Mantel test results for comparing the dissimilarity matrices of each soil type combination, values indicated R_2_ and bracketed values are P-values.**

|  | AB | TH | TN | FN | GN | SB | TM | SA |
| --- | --- | --- | --- | --- | --- | --- | --- | --- |
| **AB** |  |  |  |  |  |  |  |  |
| **TH** | **0.60 (0.01)** |  |  |  |  |  |  |  |
| **TN** | 0.28 (0.21) | 0.00 (0.34) |  |  |  |  |  |  |
| FN | 0.10 (0.28) | -0.2 (0.87) | 0.31 (0.05) |  |  |  |  |  |
| GN | 0.00 (0.52) | 0.32 (0.18) | -0.2 (0.82) | -0.0 (0.63) |  |  |  |  |
| SB | 0.46 (0.08) | **0.78 (0.01)** | 0.18 (0.28) | -0.1 (0.75) | 0.18 (0.27) |  |  |  |
| **TM** | **0.70 (0.00)** | **0.74 (0.01)** | 0.13 (0.31) | 0.04 (0.38) | 0.31 (0.14) | 0.56 (0.05) |  |  |
| **SA** | **0.73 (0.01)** | **0.79 (0.02)** | -0.1 (0.59) | -0.1 (0.79) | 0.42 (0.04) | 0.61 (0.07) | **0.7 (>0.01)** |  |


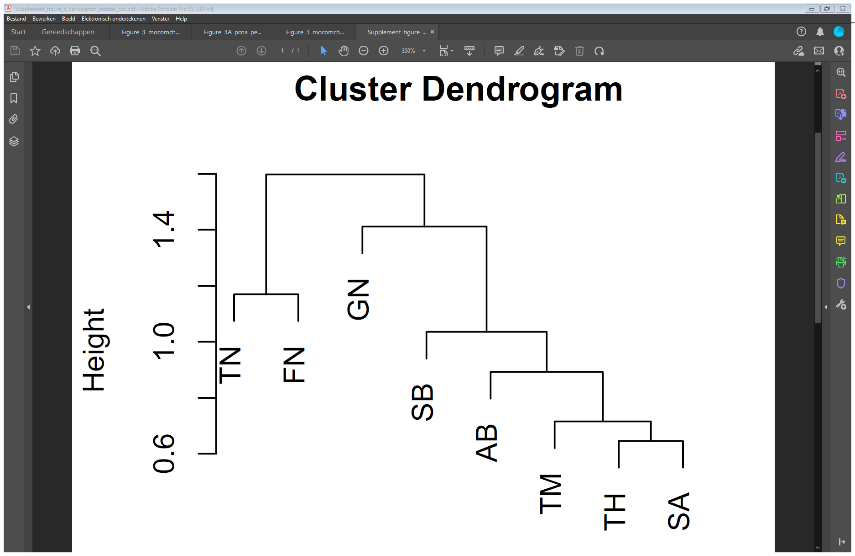


**Figure 2.** Dendrogram on the similarity of community responses of soil bacterial communities to temperature gradient at C15 sampling moment. The length of tree represents the relative dissimilarity between soil types.in community responses to temperature.
